# Multistage attention-based extraction and fusion of protein sequence and structural features for protein function prediction

**DOI:** 10.1093/bioinformatics/btaf374

**Published:** 2025-06-26

**Authors:** Meiling Liu, Shuangshuang Wang, Zeyu Luo, Guohua Wang, Yuming Zhao

**Affiliations:** College of Computer and Control Engineering, Northeast Forestry University, Harbin, 150040, China; College of Computer and Control Engineering, Northeast Forestry University, Harbin, 150040, China; College of Computer and Control Engineering, Northeast Forestry University, Harbin, 150040, China; College of Computer and Control Engineering, Northeast Forestry University, Harbin, 150040, China; College of Computer and Control Engineering, Northeast Forestry University, Harbin, 150040, China

## Abstract

**Motivation:**

Protein function prediction is important for drug development and disease treatment. Recently, deep learning methods have leveraged protein sequence and structural information, achieving remarkable progress in the field of protein function prediction. However, existing methods ignore the complex multimodal interaction information between sequence and structural features. Since protein sequence and structural information reveal the functional characteristics of proteins from different perspectives, it is challenging to effectively fuse the information from these two modalities to portray protein functions more comprehensively. In addition, current methods have difficulty in effectively capturing long-range dependencies and global contextual information in protein sequences during feature extraction, thus limiting the ability of the model to recognize critical functional residues.

**Results:**

In this study, we propose a novel framework termed Multi-stage Attention-based Extraction and Fusion model for GO prediction (MAEF-GO) based on a multistage attention mechanism to predict protein functions. MAEF-GO innovatively integrates the graph convolutional network and the graph attention network to extract protein structural features. To address the issue of modeling long-range dependencies within protein sequences, we introduce a frequency-domain attention mechanism capable of extracting global contextual relationships. Additionally, a cross-attention module is implemented to facilitate interactive fusion between protein sequence and structural modalities. Experimental evaluations demonstrate that MAEF-GO achieves superior performance compared to several state-of-the-art baseline models across standard benchmarks. Furthermore, analysis of the cross-attention weight distributions demonstrates MAEF-GO’s interpretability. It can effectively identify critical functional residues of proteins.

**Availability and implementation:**

The MAEF-GO source code can be found at https://github.com/nebstudio/MAEF-GO, an archived snapshot of the code used in this study is also available via Zenodo at https://doi.org/10.5281/zenodo.15422392.

## 1 Introduction

As an important cornerstone of life activities, proteins play a variety of key roles in organisms, including catalyzing biochemical reactions, transporting and storing substances, transmitting cell signals and immune defense, etc. (Eisenberg *et al.* 2000). Gaining deeper insight into protein functions is crucial for unraveling disease mechanisms, drug discovery and development, and personalized medicine (Yu *et al.* 2023). However, traditional experiments require expensive equipment and materials are complex, time-consuming, and have low throughput (Costanzo *et al.* 2016). Currently, in the UniProt (Bateman *et al.* 2019) database, the number of proteins that have been verified by biological experiments and obtained standard functional annotations accounts for only 0.1% of the total. At the same time, the discrepancy between unannotated sequences and annotated sequences is growing quickly (Wang *et al.* 2015, Cruz *et al.* 2017). Therefore, developing computational methods that are both efficient and accurate for predicting protein functions is highly important. Based on the type of information utilized, current methods for protein function prediction can be classified into four types: template-based, protein–protein interaction network (PPI)-based, sequence-based, and structure-based methods (Lin *et al.* 2024).

Template-based methods transfer protein functional information through the sequence alignment tool (Blast) (Buchfink *et al.* 2015) or protein family and domain alignment (FunFam) (Das *et al.* 2015). PPI-based methods take advantage of the principle that interacting proteins often have similar functions (Sharan *et al.* 2007). They predict the function of proteins by propagating labels between network nodes (Mostafavi *et al.* 2008, Mostafavi and Morris 2010) or using graph embedding techniques of PPI networks (Kulmanov *et al.* 2018, Wu *et al.* 2023). Domain, family annotation and PPI information are valuable for function prediction; however, for most proteins, this information is usually incomplete or unavailable.

Additionally, to enhance the generalization ability in predicting protein function, protein sequence-based methods have received widespread attention. For instance, DeepGOPlus (Kulmanov and Hoehndorf 2020) uses convolutional neural networks to predict protein functions based on one-hot encoding of protein sequences. Similarly, Transformer-based TALE (Cao and Shen 2021) also uses one-hot encoding for protein representation. However, one-hot encoding is only an independent representation of amino acids and lacks contextual information about the sequence and the relationship between amino acids. Therefore, excellent protein pre-trained languages such as ESM (Rives *et al.* 2021) and Prot-Trans (Elnaggar *et al.* 2022) are used to generate information-rich sequence representations (Luo *et al.* 2024). For example, DeepGO-SE (Kulmanov *et al.* 2024) predicts protein function by combining protein sequence features generated by a pre-trained protein language model with background knowledge of gene ontology. SPROF-GO (Yuan *et al.* 2023) also employs pre-trained language models to enhance protein sequence representations. Recent studies have also focused on the interpretability of the transformer model, further revealing its internal working principles in protein function prediction tasks (Wenzel *et al.* 2024).

The 3D structure of a protein is a determining factor in its function (Mitchell *et al.* 2019). Proteins with structural similarity can exhibit similar functions despite their sequences being different (Krissinel 2007). Therefore, it is crucial to use the tertiary structure information of proteins to make up for the differences in protein sequence and function. DeepFRI (Gligorijevic *et al.* 2021) applies graph convolutional networks (GCN) to protein function prediction. Graph attention network (GAT)-GO (Lai and Xu 2022) uses the structural information predicted by RaptorX (Xu *et al.* 2021) to propagate residue features through a GAT. HEAL (Gu *et al.* 2023) improves the accuracy of protein function prediction by combining hierarchical graph transformers and contrastive learning. GPSFun (Yuan *et al.* 2024) uses ESMFold (Lin *et al.* 2023) to predict structural information and extract sequence embeddings, then uses a geometric featurizer to build a protein property graph, and finally uses a graph neural network to update protein features for prediction. Struct2GO (Jiao *et al.* 2023) connects the graph representation from AlphaFold2 (Varadi *et al.* 2022) with amino acid level embedding from a pre-trained model, thereby improving the accuracy of function prediction. TAWFN (Meng and Wang 2024) trains two independent models separately to learn the graph-structured data and sequence data of proteins and further improves the robustness and accuracy of predictions by adaptively weighting and fusing the prediction results of the two models.

Although sequence- and structure-based protein function prediction methods have advanced significantly, there are still some limitations. First, they only consider one GNN model, and the advantages of using a combination of different GNN models have not been explored. Second, long-range dependencies in protein sequences are crucial in determining the functional role of specific residues and the overall protein behavior, and existing methods fail to fully capture the long-range dependencies between residues in protein sequences. In addition, when integrating one-dimensional sequence and graph structure features, existing methods ignore the interaction information between complex multimodalities.

To address the aforementioned issues, this paper proposes a novel protein function prediction model MAEF-GO. First, we fuse GCN and GAT models to learn protein structural features, where GCN captures the overall topological information of protein structure, and GAT effectively identifies and emphasizes key nodes through an adaptive attention mechanism. Second, we introduce a gated convolutional network to learn local contextual features of protein sequences; meanwhile, inspired by the excellent performance of the frequency-domain attention mechanism in capturing long-range information in natural language processing, we apply it to the protein function prediction task to effectively model the long-range dependencies between residues in protein sequences. Finally, we design a cross-attention module to achieve deep interactive fusion between protein sequence features and structural features. Experimental results show that MAEF-GO outperforms competing methods in terms of molecular function (MF), biological process (BP), and cellular component (CC). We demonstrate the effectiveness of the designed feature extraction module and fusion mechanism through ablation experiments. By analyzing the distribution of cross-attention weights, we further demonstrate the excellent interpretability of MAEF-GO and its ability to identify amino acid residues with key functions in protein sequences.

## 2 Materials and methods

### 2.1 Dataset

We adopted the same dataset as HEAL (Gu *et al.* 2023) and TAWFN (Meng and Wang 2024), which enables us to compare models fairly. The dataset is available from https://github.com/ZhonghuiGu/HEAL, which contains 36 629 protein structures (PDBset) obtained via the Protein Data Bank (Burley *et al.* 2017) database (PDBset) and 42 994 protein structures obtained via the AlphaFold2 (Varadi *et al.* 2022) database (AFset). The PDBset consists of representative protein chains annotated with at least one GO term, clustered according to a 95% sequence identity threshold. The dataset was divided into a training set (29,893 sequences), a validation set (3414 sequences) and a test set (3322 sequences) according to an 8:1:1 ratio. GO term annotations were acquired through SIFTS (Dana *et al.* 2019) and UniProtKB. The dataset covers 2752 GO terms, and the number of labels for MF terms, BP terms, and CC terms is 489, 1943, and 320, respectively. The protein structure data for each sequence are extracted from the PDB file and used to construct the protein graph. Each protein in AFset is annotated with at least one low-frequency GO term [information content (IC) > 10 in the PDBset training set].


(1) $$\mathrm{IC}({\mathrm{GO}}_{i})=-\log_{2}\left(P({\mathrm{GO}}_{i})\right)$$

Protein sequences were then clustered using MMseqs (Steinegger and Soeding 2018) with a sequence similarity threshold of 25%, where the training set contained 42 427 sequences and the test set contained 567 sequences. The sequences demonstrating sequence identity exceeding 25% with sequences in either the AFset training set or PDBset training set were deleted from the AFset test set. Lastly, the validation set was formed by randomly selecting 10% of the AFset training set.

### 2.2 Overview

**Figure 1.**
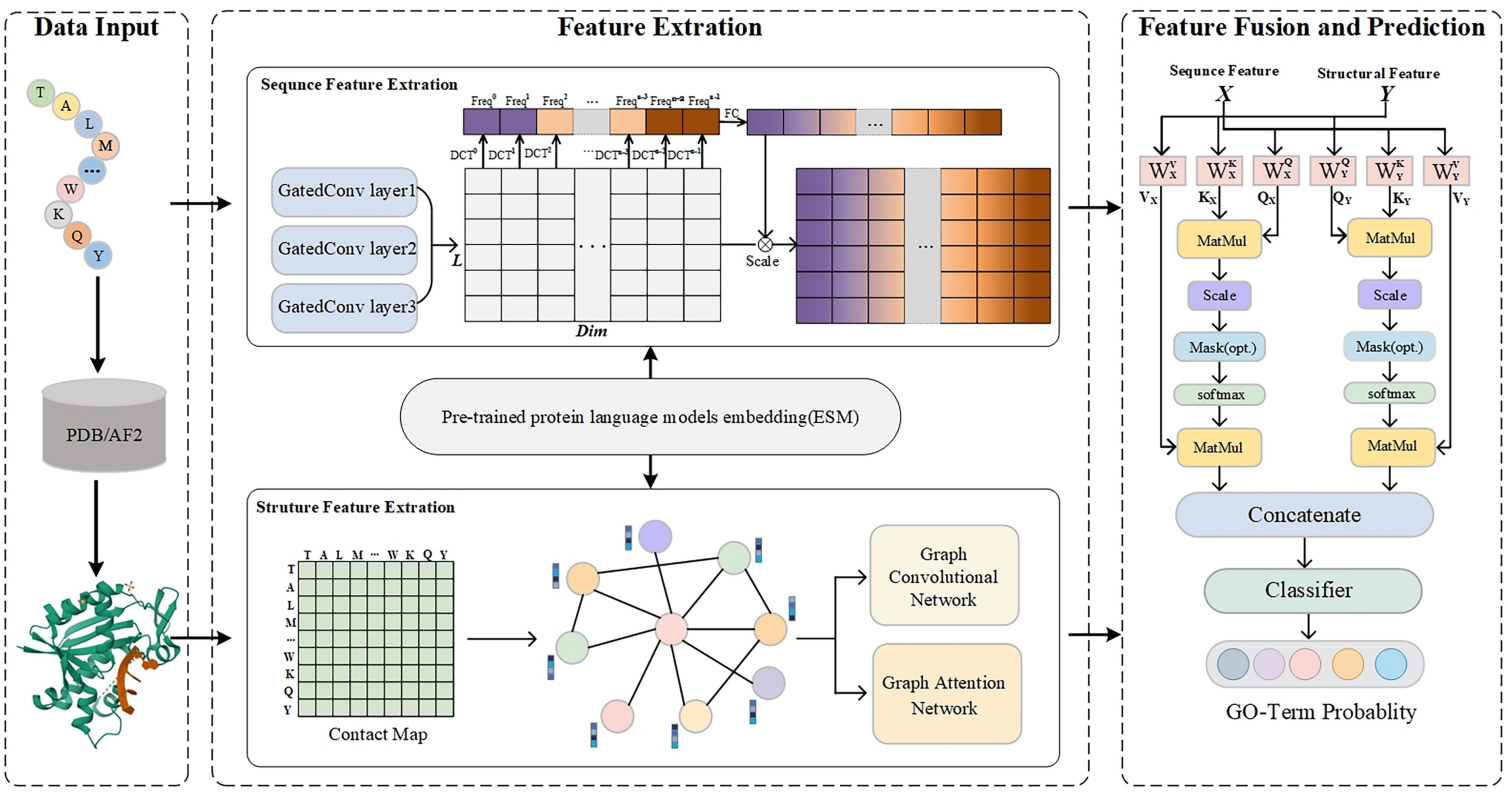
The architecture of MAEF-GO. (i) Data input module: The data input of the model is protein sequence and structure information. (ii) Feature extraction module: protein structural features are extracted by fusing GCN and GAT modules, and protein sequence features are extracted using gated convolutional neural networks and frequency-domain attention mechanism. (iii) Feature fusion and prediction module: the cross-attention mechanism is employed to realize the interactive fusion of protein structure features and sequence features, and the final GO term probability is predicted through the linear layer.

MAEF-GO uses the pre-trained ESM-1b language model to convert protein sequences into continuous vectors and uses AlphaFold2 to predict protein structures. The MAEF-GO model consists of three core components: structural feature extraction module, sequence feature extraction module, and feature fusion module. We perform feature extraction and feature fusion based on a multistage attention mechanism. The general framework of MAEF-GO is depicted in Fig. 1.

### 2.3 Amino acid embedding

In current bioinformatics research, protein language models have been widely applied. These models encode protein sequences into continuous vector representations by training from large-scale protein sequence datasets. Here, we use the ESM-1b (Rives *et al.* 2021) model to encode protein sequences into vector representations. ESM-1b is a deep contextual language model that is pre-trained on 86 billion amino acids in 250 million protein sequences through self-supervised learning to learn semantic features and patterns embedded within protein sequences. This method encodes each protein sequence into a residue-level protein sequence feature embedding ${M\in R}^{L\times 1280}$, where L refers to the protein sequence length. These features are used as subsequent inputs.

### 2.4 Protein contact maps

The protein contact map is represented by a symmetrical two-dimensional Boolean matrix, which is used to describe the spatial position of protein residues. In this paper, the structural information of proteins is extracted by constructing contact maps. First, the protein structure files in PDB format are processed using the Bio.PDB module in the Biopython package to extract the 3D atomic coordinates of each protein structure (Hamelryck and Manderick 2003). Then, if the Euclidean distance between the alpha carbon atoms of two residues in the protein structure is <10 Å, there is an edge connection between the two residues; otherwise, there is no edge connection between the two residues. The threshold of 10 Å for CA–CA contact maps was selected in accordance with DeepFRI (Gligorijevic *et al.* 2021), which demonstrated that this setting yields optimal results for CA–CA contact maps. Finally, we obtain the adjacency matrix corresponding to the protein structure $A\in R^{L\times L}$.

### 2.5 Protein sequence feature extraction module

In this module, we propose a method that combines gated convolutional networks (Dauphin *et al.* 2017) and frequency-domain attention network (Qin *et al.* 2021) to extract local and global protein sequence features. In order to capture local patterns and short-range dependencies in protein sequences effectively, we introduce the gated convolutional network. Compared to traditional convolutional neural networks, gated convolutional networks can learn features more effectively by introducing a gating mechanism, while reducing the consumption of computing resources and avoiding problems such as gradient vanishing. Each gated convolutional layer consists of a Conv1D and a gating mechanism. We use the vector M obtained from the ESM-1b model as the input to the gated convolutional network. Specifically, we design three parallel gated convolutional layers, each of which reduces the dimension from 1280 to 512. These gated convolutional layers share the same input channels but have different receptive fields. Their convolutional kernel sizes are 3, 5, and 7, respectively, to capture multi-scale features in the sequence. The outputs of the three gated convolutional layers are concatenated together through a concatenation operation to form a feature representation vector with a dimension of 1536. Subsequently, the feature vector is reduced in dimension through a fully connected (FC) layer to acquire the intermediate feature representation $D\in R^{L\times 512}$.

After local feature extraction, we further introduce a frequency-domain attention network to capture global information and long-range dependencies in protein sequences. In recent years, frequency-domain analysis has been widely used in signal processing and has produced remarkable results in the field of natural language processing. We apply it to the protein function prediction task in bioinformatics. Specifically, we use the discrete cosine transform (DCT), which is similar to the common Fourier transform. Both decompose the input signal into basis functions of different frequencies. Whereas the Fourier transform utilizes both sine and cosine functions, the DCT relies solely on cosine functions. Through DCT, the protein sequence can be decomposed into a set of frequency components, and the low-frequency coefficients mainly capture smooth long-range dependencies and global context information, while the high-frequency coefficients represent local changes and fine-grained details. By applying the attention mechanism in the frequency-domain, the model is able to adaptively emphasize or suppress features related to different frequency bands, thereby enhancing its ability to effectively capture global dependencies in protein sequences. Concretely, the input feature vector D is separated into multiple sub-vectors along the feature dimension dim, denoted as $\left[D^{1},D^{2},\ldots ,D^{n-1}\right]$, where $D^{i}\in R^{L\times \dim\prime },i\in \{0,1,\ldots ,n-1\}$, dim′=dim/n, and dim is divisible by *n*. In our implementation, we set n=512, equal to the number of feature channels, resulting in dim′=1. This choice aims to maintain the independence of each feature channel, thereby preserving their biological semantics after the frequency-domain transformation. For each part $D^{i}$, DCT is applied to extract the frequency-domain feature ${\mathrm{Freq}}^{i}$:


(2) $${\mathrm{Freq}}^{i}=\mathrm{DCT}(D^{i}).$$

Next, by concatenating all the sub-parts, we can obtain the complete frequency-domain feature vector Freq:


(3) $$\mathrm{Freq}=\mathrm{cat}([{\mathrm{Freq}}^{0},{\mathrm{Freq}}^{1},\ldots ,{\mathrm{Freq}}^{n-1}]).$$

Finally, the frequency-domain attention weight is calculated through the FC layer and the sigmoid activation function, and the frequency-domain information is used to enhance the expression ability and improve the ability to capture long-range dependencies. After being processed by the frequency-domain attention network, we get the protein sequence feature vector X∈L×512. The frequency-domain attention weight can be computed using the following formula:


(4) X=sigmoid(FC(Freq)).

### 2.6 Protein structure feature extraction module

In this module, we use GNN architectures to extract protein structural features. A graph representation can be used for protein structures, in which amino acid residues correspond to nodes in the graph and contact relationships between residues correspond to edges of the graph. The proposed GNN is an intermediate fusion of two types of GNN architectures. Specifically, we designed a GCN module containing three GCN layers (Kipf and Welling 2017) and a GAT module containing three GAT layers (Veličković *et al.* 2017) which are cascaded in parallel to process the input protein structure graph. Specifically, the output node features from the GCN and GAT modules are concatenated along the feature dimension to achieve feature fusion. After the graph fusion network, we obtain the protein structure feature vector Y∈L×512. During the message passing process, the node feature update functions of GCN and GAT are as follows:


(5) $$H^{l+1}=\mathrm{ReLU}({\hat{D}}^{-0.5}\hat{A}{\hat{D}}^{-0.5}H^{\left(l\right)}W^{\left(l\right)}),$$

where$\;\hat{A}=A+I$ is the adjacency matrix with added self-loops, $\hat{D}$ is the degree matrix of the node, $H^{\left(l\right)}$ is the feature representation matrix of modes at the lth, $W^{\left(l\right)}$ is the learnable weight matrix at lth layer, $H^{l+1}$ is the matrix of node feature representations at the (l+1)th layer for all nodes in the graph, and ReLU(.) is the Rectified Linear Unit activation function.


(6) $$h_{i}^{´}=\;\mathrm{ReLU}(\underset{j\in N_{i}}{\sum }\alpha_{ij}Wh_{j}),$$

where $N_{i}$ denotes the set of neighboring nodes of node i, W is the learnable weight matrix used to linearly transform the input feature $h_{j}$, $h_{i}^{´}$ is the updated feature vector of node i after the GAT layer transformation, and the normalized attention coefficient between node i and node j is calculated as follows:


(7) $$\alpha_{ij}=\frac{\exp(e_{ij})}{\sum_{k\in N_{i}}e\mathrm{xp}(e_{ik})},$$

where $e_{ij}$ and $e_{ik}$ are the attention coefficients between node i and its neighbor nodes j and k.

### 2.7 Cross-feature fusion module

Protein structure and sequence features contain their own interesting information. If they are directly combined, the relationship between them will be ignored. The cross-attention mechanism (Vaswani *et al.* 2017) is capable of constructing explicit interactions between two independent inputs, thus exploiting their relevance. Inspired by this property, we use the cross-attention mechanism to achieve a deep fusion of sequence features and structural features. The cross-attention network is defined as follows:


(8) $$\begin{array}{c} {\;\mathrm{CrossAtt}}_{\mathrm{seq}}=\mathrm{softmax}\;(\frac{Q_{X}K_{X}^{T}}{\sqrt{d_{k_{X}}}})V_{X} \end{array},$$

(9) $${Q_{X}=XW_{X}^{Q},K}_{X}=YW_{X}^{K},V_{X}=YW_{X}^{V},$$

(10) $${\;\mathrm{CrossAtt}}_{\mathrm{stru}}=\mathrm{softmax}\;(\frac{Q_{Y}K_{Y}^{T}}{\sqrt{d_{k_{Y}}}})V_{Y},$$

(11) $${Q_{Y}=YW_{Y}^{Q},K}_{Y}=XW_{Y}^{K},V_{Y}=XW_{Y}^{V},$$

where X and Y represent the embedding matrices of protein sequence and structural features, respectively. In the cross-attention mechanism for protein sequences, the query matrix $Q_{X}$ is calculated from X, the key matrix $K_{X}$ and the value matrix $V_{X}$ are calculated using the opposite inputs. These six matrices $W_{X}^{K}$, $W_{X}^{V}$, $W_{X}^{Q}\;W_{Y}^{K}$, $W_{Y}^{V}$, $W_{Y}^{Q}$ are learnable parameters and $d_{k_{X}}$ and $d_{k_{Y}}$ represent the dimensions of $K_{X}$ and $K_{Y}$, respectively. In this study, we adopt a multihead cross-attention mechanism with two attention heads, resulting in the concatenation of outputs from two parallel cross-attention layers.

### 2.8 Model training

We employed PyTorch (version 1.9.0) and the PyTorch Geometric library (Fey and Lenssen 2019) for the implementation of our model, which was trained via the Adam optimizer (Kingma and Ba 2015) and the binary cross-entropy loss function, using a learning rate of 1e−4, a batch size of 64, and for 100 epochs. To avoid overfitting, we implemented an early stopping strategy monitored by the validation set, setting the patience to five epochs. During training, the model with the lowest loss on the validation set was retained as the final model. All experiments were performed on a single NVIDIA RTX 3090 GPU (24 GB memory).

### 2.9 Baseline methods

We conducted extensive experiments to compare MAEF-GO with several benchmark methods. For details, see Supplementary Material S1. These methods include Blast (Buchfink *et al.* 2015), FunFams (Das *et al.* 2015), DeepGO (Kulmanov *et al.* 2018), DeepGOPlus (Kulmanov and Hoehndorf 2020), DeepFRI (Gligorijevic *et al.* 2021), GAT-GO (Lai and Xu 2022), GPSFun (Yuan *et al.* 2024), DeepGO-SE (Kulmanov *et al.* 2024), HEAL (Gu *et al.* 2023), and TAWFN (Meng and Wang 2024). Specifically, Blast and FunFams are protein function annotation methods based on sequence similarity and domain structure, respectively; DeepGO uses CNN to combine protein sequence and network features for protein function prediction; DeepGOPlus predicts protein function from sequence alone; DeepFRI and GAT-GO combine protein sequence and structure information to propagate residue features based on GCN and GAT, respectively; GPSFun uses a geometric featurizer to build a protein property graph; DeepGO-SE generates protein embeddings using a pre-trained language model; HEAL is a hierarchical graph transformer approach that integrates sequence and structure information; and TAWFN is an integrated learning framework that combines CNN and GCN, which fuses the prediction results of the two networks through adaptive weights.

### 2.10 Evaluation metrics

In this study, we used CAFA (Radivojac *et al.* 2013) evaluation metrics $F_{\max}$, AUPR and $S_{\min}$ to measure the performance of different methods. $F_{\max}$ represents the maximum value of the F1 score at different thresholds. The F1 value represents the harmonic average of precision and recall. AUPR is based on the area under the precision-recall curve and is used to evaluate the prediction performance of models with high class imbalance. $S_{\min}\;$ is based on the minimum value of the semantic distance, and it assesses semantic consistency between the predicted function and actual function. The detailed calculation method of each indicator is provided in the Supplementary Material S2.

## 3 Results

### 3.1 The comparison of MAEF-GO and baseline models

We compared MAEF-GO with several existing methods, including Blast, FunFams, DeepGO, DeepFRI, GAT-GO, GPSFun, DeepGO-SE, HEAL, and TAWFN. We assessed the performance of MAEF-GO on the PDBset test set for MF, BP, and CC prediction tasks, with a 95% sequence identity threshold to the training set. The results are shown in Table 1. In the three prediction tasks of MF, BP, and CC, MAEF-GO achieved AUPR scores of 0.758, 0.438, 0.530; $F_{\max}$ scores of 0.787, 0.652, 0.720; and $S_{\min}$ scores of 0.298, 0.461, 0.426, respectively. Obviously, MAEF-GO significantly outperformed other comparison methods in all three types of GO tasks. This is attributed to the effectiveness of our feature extraction module designed for protein sequence and structural information, respectively, as well as the proposed multimodal feature fusion module. Compared to other popular models, MAEF-GO can capture local and global features of protein sequences and structures more adequately and achieve interactive fusion of multimodal information.

**Table 1.** Performance comparison of different methods on PDBset test set. Bold numbers denote the best results.

Method	AUPR			$F_{\max}$			$S_{\min}$		
	MF	BP	CC	MF	BP	CC	MF	BP	CC
Blast	0.136	0.067	0.096	0.326	0.336	0.443	0.643	0.662	0.632
FunFams	0.370	0.256	0.265	0.573	0.498	0.640	0.542	0.58	0.512
DeepGO	0.391	0.189	0.258	0.576	0.500	0.589	0.475	0.578	0.553
DeepFRI	0.495	0.265	0.274	0.627	0.546	0.617	0.432	0.543	0.530
GAT-GO	0.660	0.381	0.479	0.633	0.492	0.547	0.437	0.521	0.466
GPSFun	0.601	0.203	0.309	0.745	0.456	0.630	0.339	0.564	0.508
DeepGO-SE	0.495	0.233	0.423	0.654	0.566	0.636	0.435	0.53	0.481
HEAL	0.691	0.337	0.467	0.747	0.595	0.687	0.342	0.509	0.458
TAWFN	0.718	0.385	0.488	0.762	0.628	0.693	0.326	0.483	0.454
MAEF-GO	**0.758**	**0.438**	**0.530**	**0.787**	**0.652**	**0.720**	**0.298**	**0.461**	**0.426**

### 3.2 Ablation study

To validate the effectiveness of our designed feature extraction module and feature fusion module, we conducted ablation experiments. Specifically, four ablation models were designed: (i) MAEF-GO w/o GCN: remove the GCN module. (ii) MAEF-GO w/o GAT: remove the GAT module. (iii) MAEF-GO w/o FDA: remove the frequency-domain attention module. (iv) MAEF-GO w/o CA: remove the cross-attention module. The experimental results are shown in Table 2.

**Table 2.** Ablation experiment results of MAEF-GO on PDBset test set. Bold numbers denote the best results.

Method	AUPR			$F_{\max}$			$S_{\min}\;$		
	MF	BP	CC	MF	BP	CC	MF	BP	CC
MAEF-GO	**0.758**	**0.438**	**0.530**	0.787	**0.652**	**0.720**	**0.298**	**0.461**	**0.426**
MAEF-GO w/o GCN	0.745	0.427	0.523	0.778	0.649	0.715	0.302	0.469	0.431
MAEF-GO w/o GAT	0.748	0.425	0.523	0.780	0.646	0.713	0.309	0.469	0.434
MAEF-GO w/o FDA	0.754	0.424	0.520	**0.789**	0.642	0.706	0.301	0.468	0.437
MAEF-GO w/o CA	0.748	0.417	0.513	0.781	0.644	0.711	0.309	0.477	0.438

In the structural feature extraction module, removing either GCN or GAT results in decreased model performance, indicating that the combination of GCN and GAT can more comprehensively extract structural features of proteins. Because GCN is good at capturing the topological relationship between nodes from a global perspective, and GAT refines local information by assigning different weights to different neighbor nodes, the combination of the two can more accurately capture the complex structural features of proteins. In addition, when we remove the frequency-domain attention network from the protein sequence feature extraction module, the performance of most evaluation metrics declines. We observed that, although the performance on the MF ontology remained nearly unchanged, the inference time of the model did not increase significantly. Specifically, we randomly selected 500 proteins from the PDBtest test set and measured inference time on an NVIDIA RTX 3090 GPU with a batch size of 64. The results showed that the inference time with the FDA module was 14.86 s, while it was 14.35 s without the FDA module. The frequency-domain attention network captures long-range dependency information in protein sequences through frequency-domain analysis, which enhances the model’s ability to model global features of the sequence. This introduces a novel and effective method for the field of bioinformatics to handle complex long-range information features of protein sequences. In terms of feature fusion, when we remove the cross-attention module and directly fuse sequence and structural features, it significantly reduces the prediction performance of the model, clearly demonstrating the importance of multimodal interaction. Overall, our proposed method significantly enhances the performance of protein function prediction. To further evaluate the effectiveness of the frequency-domain attention mechanism, we replaced the frequency-domain attention module with a Transformer-based self-attention module and conducted experiments while keeping other settings unchanged. Experimental results show that the frequency-domain attention mechanism performs better in this task. See Table 1, available as supplementary data at *Bioinformatics* online for the relevant experimental results.

### 3.3 Generalization experiment of MAEF-GO

**Figure 2.**
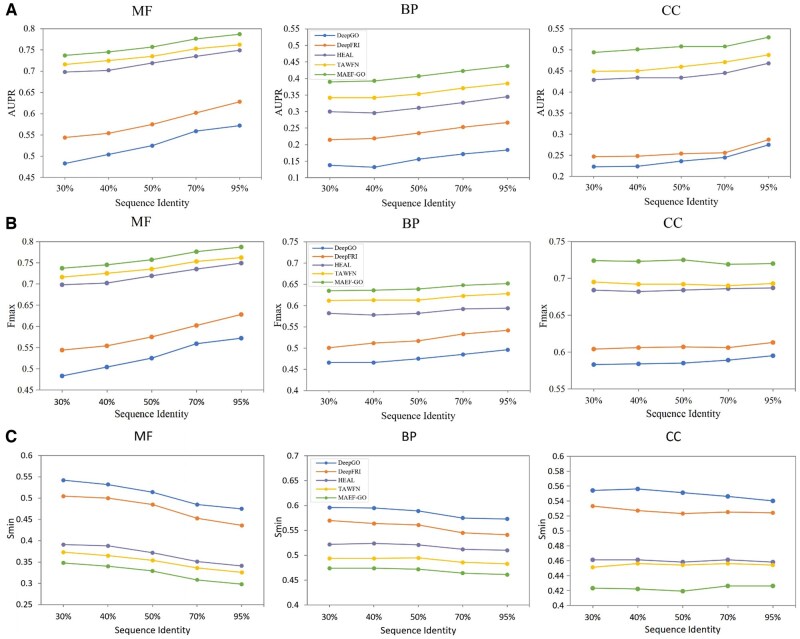
(A) AUPR, (B) $F_{\max}$, and (C) $S_{\min}\;$for different methods with varying sequence identities on PDBset test set.

To evaluate MAEF-GO’s generalization ability, we performed tests on the PDBset test set at five homology thresholds (30%, 40%, 50%, 70%, and 95%). These thresholds represent the highest degree of similarity between the test sequence and the training dataset, which can more accurately evaluate the generalizability of the model on new sequences. The number of proteins evaluated at each threshold can be found in Table 2, available as supplementary data at *Bioinformatics* online. In other fields, like protein structure prediction (AlQuraishi 2019), this evaluation method is widely employed. We compared MAEF-GO with DeepGO, DeepFRI, HEAL, and TAWFN, and the results are shown in Fig. 2. MAEF-GO significantly outperformed other methods under all five threshold conditions. In addition, the performance of MAEF-GO on the low-homology test set is comparable to or better than that of other methods under high homology conditions. This shows that compared with TAWFN’s method of training two independent models to extract protein graph structure and sequence features, respectively, and then fusing them through adaptive weights, our method can capture protein features more comprehensively. For details, see Tables 3–5, available as supplementary data at *Bioinformatics* online.

We then evaluated the performance of MAEF-GO on GO terms with different specificity levels, and divided the GO terms in the PDBset test set into three groups based on their IC value. A higher IC value indicates greater functional specificity, appears less frequently in the dataset, and increases prediction difficulty for the model. The results show that MAEF-GO achieves higher AUPR for low, moderate, and high specificity GO terms. See Fig. 1, available as supplementary data at *Bioinformatics* online for the relevant experimental results.

### 3.4 Performance of MAEF-GO on AlphaFold2 predicted structures

**Figure 3.**
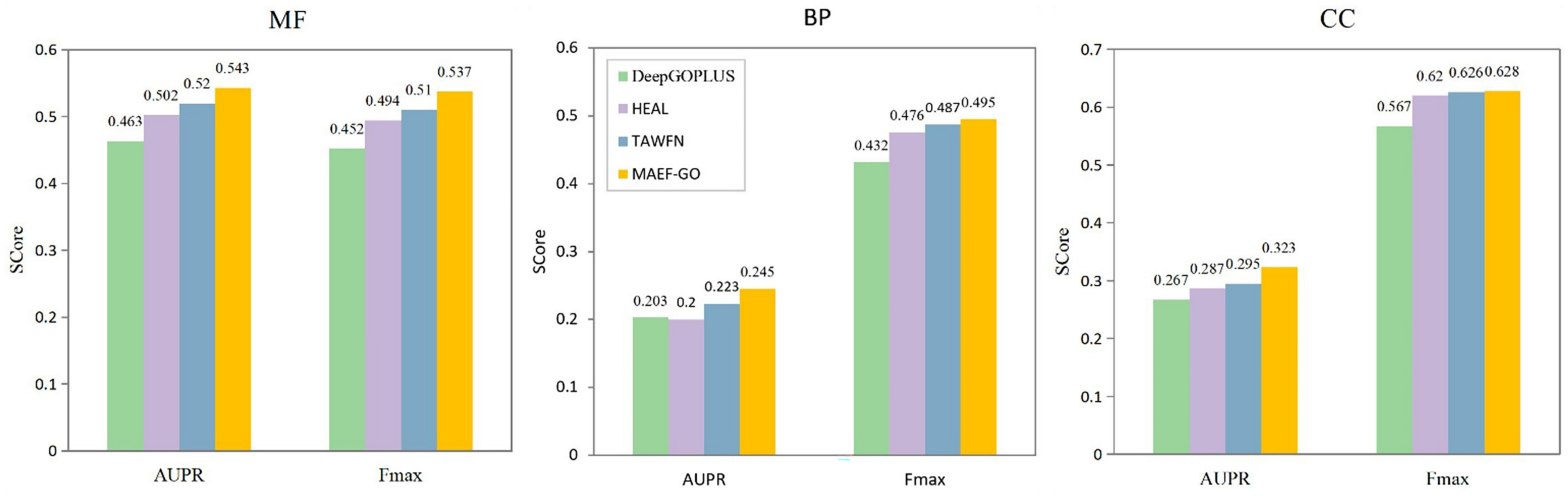
$F_{\max}$ and AUPR performance for different methods on AFset test set.

A more realistic application scenario for MAEF-GO is the prediction of the biological functions of proteins without experimentally determined structures and similar sequence annotations. To verify the effectiveness of MAEF-GO, we evaluated it on the AFset test set. We compared the performance of MAEF-GO with methods such as DeepGOPlus, HEAL, and TAWFN. The experimental results are shown in Fig. 3. MAEF-GO outperforms DeepGOPlus, which is based only on sequences, and HEAL, which is based only on GNNs, and also achieves better performance than TAWFN, which uses adaptive weight fusion of GCN and CNN network prediction results. These results show the effectiveness of MAEF-GO in more practical application scenarios.

To further evaluate whether model performance improved with more functional annotation data included. We construct low-homology protein dataset using the MMseqs2 tool to obtain 10 269 protein sequences from the AlphaFold database that share less than 25% sequence identity with both the original training and test sets. These proteins were divided randomly into training and validation sets at a 9:1 ratio. After incorporating these low-homology proteins into the training set, we re-evaluated model performance on the AFset test set. As shown in Table 6, available as supplementary data at *Bioinformatics* online, despite the increase in training data size, the model’s performance on the test set did not improve significantly.

To better understand this outcome and validate the functional annotation representativeness of the original dataset, we further evaluated MAEF-GO using these 10 269 low-homology proteins as an independent test set (not added to the training dataset, and referred to as ExtSet). The results, presented in Table 6, available as supplementary data at *Bioinformatics* online, show that MAEF-GO achieves similar performance on both the AFset and ExtSet test sets. Considering that the original training dataset (Meng and Wang 2024) has already been subject to rigorous homology control and possesses a relatively comprehensive range of functional diversity. Hence, the newly added low-homology proteins may not expand the functional space captured by the training data as expected.

Although the training set contains approximately 68 078 sequences, which is relatively limited compared to the 5500 000 annotated functional proteins available in UniProt, we speculate that incorporating the entire UniProt database into training may not lead to substantial model performance improvements. This is supported by the observed stability in model accuracy after simulating low-homology expansion. Additionally, excessive training data may introduce redundancy, whereby the majority-class functions dominate the learning process and suppress the representation of minority-class functions. It is also possible that the model architecture has a finite capacity to benefit from increased data volume.

Generally, our study presents a relatively generalizable GO function prediction model. Future work may benefit from exploring the construction of datasets that are more functionally diverse while maintaining strict control over redundancy, as well as improvements in model architecture or training strategies to enhance the model performance in annotating minority-class functions.

### 3.5 Identify critical residues based on attention mechanism

**Figure 4.**
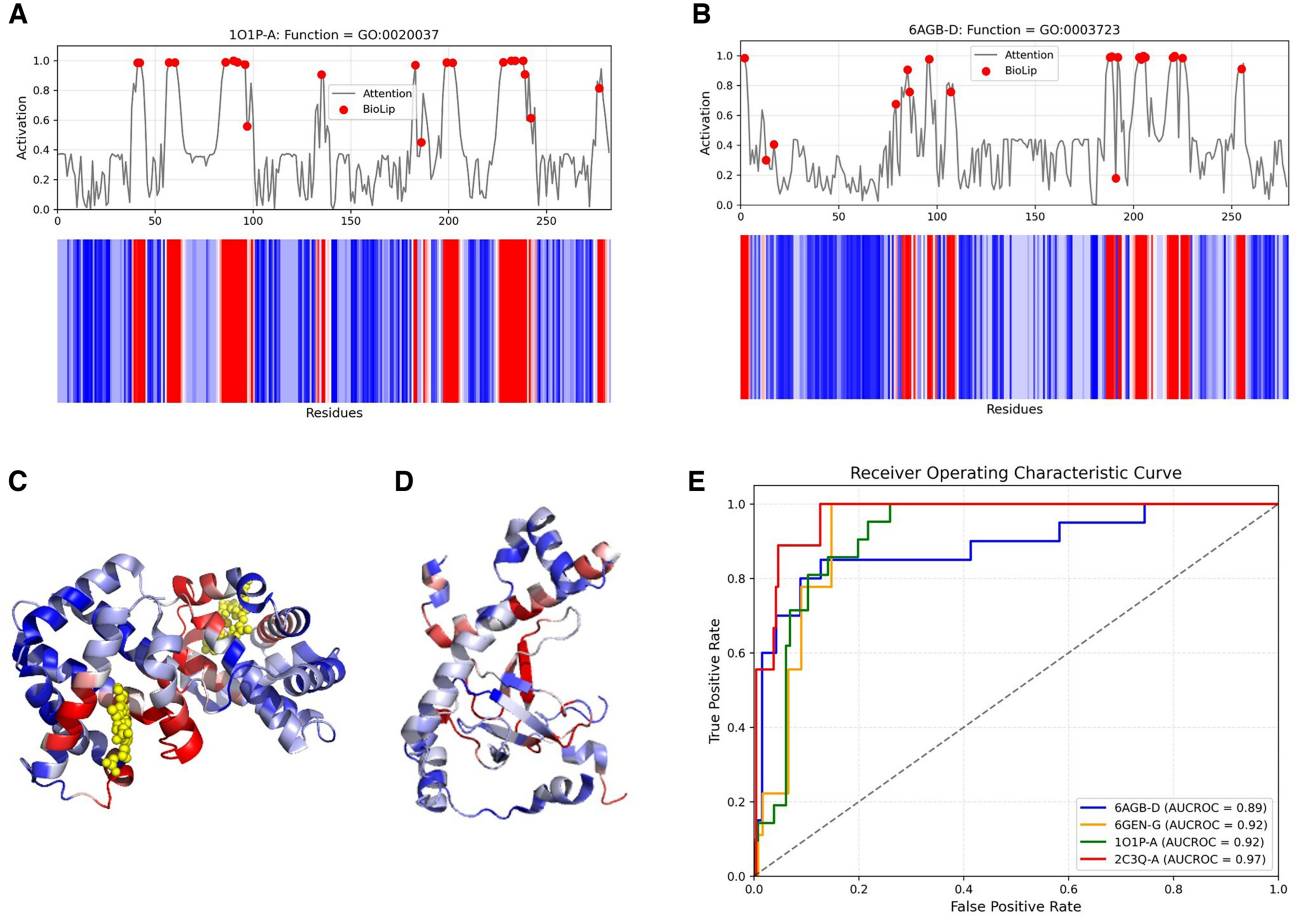
MAEF-GO uses attention weights to visualize functional residues. (A) Attention weights (GO: 0020037) for deoxy hemoglobin (PDB: 1O1P, chain A) with heme binding function. (B) Attention weights (GO: 003723) for yeast ribonuclease P (PDB: 2PE5, chain B) with RNA binding function. (C) and (D) Projection of heatmaps onto the 3D structures of the corresponding proteins. (E) ROC curves of residues identified by attention weights and binding sites obtained from the BioLip database.

According to previous studies (Lichtarge *et al.* 1996), spatially clustered functional residues play a key role in protein function. In order to evaluate the ability of MAEF-GO in predicting residue positions related to protein function, we analyzed the attention weights assigned in its cross-attention module to identify key residues that contribute more to the target function. In Fig. 4A, we show a heatmap of the average attention weights, where red indicates a larger contribution, and blue indicates a smaller contribution. The gray line indicates the distribution of attentional weights across the protein sequence and the red dots indicate experimentally verified protein-ligand binding sites collected from the BioLip (Yang *et al.* 2013) database. This shows that MAEF-GO is able to correctly identify functional sites related to heme binding (GO: 0020037) in deoxy hemoglobin (PDB: 1O1P; chain A). In addition, in Fig. 4B, we show another case in which MAEF-GO accurately identified the majority of the binding sites of yeast ribonuclease P (PDB: 6AGB; chain D) with RNA binding function (GO: 003723). We projected the attention weight heatmaps of 1O1P and 6AGB onto their corresponding protein 3D structures (as shown in Fig. 4C and D). The results show that residues close to the ligand have a greater contribution to the function of the ligand-binding protein. Heatmaps of other cases and corresponding protein tertiary structure visualizations are shown in Supplementary Material S4.

We quantified the ability of MAEF-GO to recognize critical residues by means of receiver operating characteristic (ROC) curves. Specifically, we computed the area under the curve (AUC) for the following proteins: deoxy hemoglobin (PDB: 1O1P; chain A), yeast ribonuclease P (PDB: 6AGB; chain D), human glutathione-S-transferase (PDB: 2C3Q; chain A), and DNA polymerase (PDB: 6GEN; chain G), corresponding to heme binding (GO: 0020037), RNA binding (GO: 003723), glutathione transferase activity (GO: 0004364), and DNA binding (GO: 0003677) functions, respectively. As shown in Fig. 4E, the ROC curve and the calculated AUC show that MAEF-GO can focus more on residues significantly contributing to protein function annotation, and has an excellent ability to capture functional binding residues.

## 4 Conclusion

In this study, we proposed a novel deep learning model, MAEF-GO, to predict protein functions. Our model strategically extracts features from amino acid sequences and spatial structures of proteins and interactively fuses them. Specifically, in terms of sequence feature extraction, MAEF-GO introduces a frequency-domain attention mechanism to map protein sequence information to the frequency-domain space. By adaptively allocating frequency-domain attention weights, the model can distinguish and emphasize different frequency components, thereby more effectively capturing long-range dependencies and global contextual information in the sequence. In terms of structural feature extraction, the model integrates GCNs and GATs to fully mine the overall topological features and local key node information of the three-dimensional structure of proteins. In addition, through the cross-attention mechanism, the sequence and structural features of proteins are effectively integrated, realizing the deep interactive fusion of multimodal information. This significantly enhances the ability to predict protein function.

The experimental results of this method on two benchmark datasets show that MAEF-GO outperforms other advanced models in prediction performance. Ablation experiments validated the effectiveness of each module in the model; MAEF-GO also shows robustness and effectiveness on novel proteins with low sequence identity. Furthermore, by analyzing the distribution of cross-attention weights, MAEF-GO provides important biological significance explanations: MAEF-GO can analyze the attention weights of single amino acid residues to identify functional sites, and explains that the key residues with high attention weights identified by MAEF-GO are highly consistent with the experimental data in the BioLip database. Overall, MAEF-GO represents a promising approach for protein function prediction and functional site identification using deep learning techniques, which can help researchers better understand disease mechanisms or conduct drug design.

Although MAEF-GO performs well, its representation of protein structures remains insufficiently comprehensive. With the advancement of AlphaFold3 (Abramson *et al.* 2024), obtaining high-quality protein structural information has become increasingly efficient. In future work, we aim to further enhance the graph structure representation, for example by employing heterogeneous graphs, to more comprehensively characterize protein structural features and the complex interactions among amino acids. In addition, the frequency-domain attention module has not yet utilized biological properties such as protein domains. Future work will explore biologically guided feature partitioning to improve the accuracy and interpretability of model predictions.

## Supplementary Material

btaf374_Supplementary_Data

## Data Availability

The MAEF-GO source code and data can be found at https://github.com/nebstudio/MAEF-GO.
