# Supplementary material for: Multistage attention-based extraction and fusion of protein sequence and structural features for protein function prediction
Source: Bioinformatics. 2025 Jun 26;41(7):btaf374. doi: 10.1093/bioinformatics/btaf374 (PMC12289230; doi:10.1093/bioinformatics/btaf374)
Supplement: btaf374_Supplementary_Data [file btaf374_supplementary_data.docx]

**1 Baseline methods**

BLAST: A basic sequence alignment tool that identifies similar regions between sequences through a local alignment algorithm. In protein function prediction, BLAST compares protein sequences in a test dataset with those in a training dataset and assigns functional annotations to proteins based on similarity scores.

FunFams: A domain-based protein function annotation method. For each protein sequence, the HMMER3 tool is used to scan the CATH FunFam database. Based on the matching score between the test sequence and FunFam, the functional label corresponding to the highest-scoring FunFam is transferred to the test sequence. The prediction score is the frequency of occurrence of the functional category.

DeepGO: A one-dimensional convolutional neural network (CNN) is used to extract protein sequence features and integrate the knowledge graph embedding features obtained from the protein-protein interaction network. A deep learning method that uses the GO hierarchy as background information.

DeepFRI: A deep learning method utilizing graph convolutional network (GCN). The protein contact graph is constructed using structural information obtained from PDB and SWISS-MODEL, and a protein language model is trained to obtain protein sequence embeddings. Combine protein sequence and structural features and input them into GCN to predict protein function.

GAT-GO: Compared with DeepFRI, GAT-GO uses a graph attention network (GAT) instead of a traditional GCN, and predicts residue-protein contacts through RaptorX instead of the natural contacts used by DeepFRI. GAT-GO predicts protein function by feeding the protein contact graph and CNN-generated sequence representation into GAT.

GPSFun: A multi-task learning model that uses a large language model to predict structural information and extract sequence features, and finally uses a graph neural network to update protein features for prediction.

DeepGO-SE: The protein prediction performance is improved by using the pre-trained language model ESM-2 to generate protein sequence features and combining background knowledge from GO and protein-protein interactions.

HEAL: A contrastive learning-assisted protein function prediction method based on GCN. Utilizing sequence and structural information, node aggregation and graph pooling are performed by introducing a hierarchical graph transformer. In addition, HEAL expands the dataset based on DeepFRI by combining the protein structure predicted by ALphaFpld2.

TAWFN: A deep learning model that utilizes ensemble learning to achieve protein function prediction. TAWFN trains two independent sub-models to learn the graph structure data and sequence data of proteins respectively, and integrates the prediction results of the two models through a weight adaptive fusion algorithm.

**2.Evaluation metrics**

1. $\mathrm{Fmax}$

$Fmax=\max_{t}\frac{2AvgPr \left( t \right)*AvgRc \left( t \right)}{AvgPr \left( t \right)+AvgRc \left( t \right)}$ (1)

$AvgPr \left( t \right)=\frac{1}{m\left( t \right)}*\sum_{i=1}^{m\left( t \right)} pr_{i}\left( t \right)$ (2)

$AvgRc \left( t \right)=\frac{1}{n}*\sum_{i=1}^{n} rc_{i}\left( t \right)$ (3)

$Fmax$ is the maximum F1 value at different thresholds $t$ (Formula 1). $AvgPr(t)$ and $AvgRc(t)$ are the average precision and average recall at threshold $t$, respectively (Formula 2 and Formula 3). ${pr}_{i}(t)$ and $rc_{i}(t)$ denote the precision and recall of protein $i$ under threshold $t$. The threshold $t$ ranges from 0 to 1 with a step size of 0.01. $n$ represents the total overall count of proteins in the test set, and $m(t)$ represents the number of proteins have one or more functional annotation at threshold $t$.

2．AUPR

$\text{macro-AUPR}\text{=}\text{∫}\text{ }Avg{Pr}^{'} (t)*d(AvgRc^{'}(t))$ (4)

$Avg {Pr}^{'} \left( t \right)=\frac{1}{f\left( t \right)}\sum_{j=1}^{n} pr_{j}^{'}\left( t \right)$ (5)

$AvgRc^{'}\left( t \right)=\frac{1}{f\left( t \right)}\sum_{j=1}^{n} rc_{j}^{'}\left( t \right)$ (6)

$\text{macro-AUPR}$ denotes the area under the curve of $Avg{Pr}^{'}(t)$ and $AvgRc^{'}(t)$ (Formula 4). Unlike AvgPr(t) and AvgRc(t), which calculate the average value of each protein, $Avg{Pr}^{'}(t)$ and $AvgRc^{'}(t)$ denote the average precision and average recall for each function at threshold $t$ (Formula 5 and Formula 6).

3. $\mathrm{Smin}$

$Smin=\min_{t}\sqrt{ru(t)^{2}+mi(t)^{2}}$ (7)

$ru(t)=\frac{1}{n}\sum_{i=1}^{n} \underset{c\in T_{i}-P_{i}(t)}{\sum}IC(l)$ (8)

$mi(t)=\frac{1}{n}\sum_{i=1}^{n} \underset{c\in P_{i}-T_{i}(t)}{\sum}IC(l)$ (9)

$IC(l)=-{log}_{2}(P(l))$ (10)

$Smin$ is used to calculate the minimum semantic distance between the predicted function and the actual function at different thresholds (Formula 7). $ru(t)$ denotes the average residual uncertainty (Formula 8), and $mi(t)$ represents the average error information (Formula 9), where $T_{i}$ denotes the true annotation of protein $i$. $IC(l)$ denotes the information content of quantified function $l$ (Formula 10), where $P(l)$ denotes the probability of occurrence of function $l$.

**3.Result**

Table S1. Performance of MAEF-GO-Trans (MAEF-GO with frequency-domain attention replaced by transformer-based self-attention) on the PDBset test set.

| **Method** | **AUPR** | | |  | $\mathbf{F}_{\mathbf{max}}$ | | |  | $\mathbf{S}_{\mathbf{min}}$ | | |
| --- | --- | --- | --- | --- | --- | --- | --- | --- | --- | --- | --- |
|  | **MF** | **BP** | **CC** |  | **MF** | **BP** | **CC** |  | **MF** | **BP** | **CC** |
| MAEF-GO-Trans | 0.746 | 0.425 | 0.518 |  | 0.773 | 0.639 | 0.708 |  | 0.310 | 0.470 | 0.439 |

Table S2. Number of PDBset test set proteins under different sequence identity thresholds to the training set.

| threshold | 30% | 40% | 50% | 70% | 95% |
| --- | --- | --- | --- | --- | --- |
| Proteins | 1716 | 1936 | 2198 | 2732 | 3414 |

Table S3. AUPR of MAEF-GO and other methods on the PDBset test set at five homology thresholds.

| AUPR of MF ontology | | | | | |
| --- | --- | --- | --- | --- | --- |
| Model | <30% | <40% | <50% | <70% | <95% |
| DeepGO | 0.303 | 0.324 | 0.348 | 0.384 | 0.395 |
| DeepFRI | 0.426 | 0.447 | 0.461 | 0.489 | 0.503 |
| HEAL | 0.638 | 0.641 | 0.663 | 0.681 | 0.698 |
| TAWFN | 0.672 | 0.68 | 0.691 | 0.701 | 0.718 |
| **MAEF-GO** | **0.718** | **0.725** | **0.734** | **0.740** | **0.758** |
| AUPR of BP ontology | | | | | |
| Model | <30% | <40% | <50% | <70% | <95% |
| DeepGO | 0.138 | 0.132 | 0.156 | 0.172 | 0.184 |
| DeepFRI | 0.215 | 0.219 | 0.235 | 0.253 | 0.267 |
| HEAL | 0.300 | 0.296 | 0.311 | 0.327 | 0.345 |
| TAWFN | 0.342 | 0.342 | 0.353 | 0.371 | 0.385 |
| **MAEF-GO** | **0.390** | **0.393** | **0.407** | **0.423** | **0.438** |
| AUPR of CC ontology | | | | | |
| Model | <30% | <40% | <50% | <70% | <95% |
| DeepGO | 0.223 | 0.224 | 0.236 | 0.245 | 0.275 |
| DeepFRI | 0.247 | 0.248 | 0.254 | 0.256 | 0.287 |
| HEAL | 0.429 | 0.434 | 0.434 | 0.445 | 0.468 |
| TAWFN | 0.449 | 0.45 | 0.46 | 0.471 | 0.488 |
| **MAEF-GO** | **0.494** | **0.501** | **0.508** | **0.508** | **0.530** |

Table S4. Fmax of MAEF-GO and other methods on the PDBset test set at five homology thresholds.

| $F_{\max}$of MF ontology | | | | | |
| --- | --- | --- | --- | --- | --- |
| Model | <30% | <40% | <50% | <70% | <95% |
| DeepGO | 0.483 | 0.504 | 0.525 | 0.559 | 0.572 |
| DeepFRI | 0.544 | 0.554 | 0.575 | 0.602 | 0.628 |
| HEAL | 0.698 | 0.702 | 0.719 | 0.735 | 0.749 |
| TAWFN | 0.716 | 0.725 | 0.735 | 0.753 | 0.762 |
| **MAEF-GO** | **0.737** | **0.745** | **0.757** | **0.776** | **0.787** |
| $F_{\max}$of BP ontology | | | | | |
| Model | <30% | <40% | <50% | <70% | <95% |
| DeepGO | 0.466 | 0.466 | 0.475 | 0.485 | 0.496 |
| DeepFRI | 0.501 | 0.512 | 0.517 | 0.533 | 0.542 |
| HEAL | 0.582 | 0.578 | 0.582 | 0.592 | 0.594 |
| TAWFN | 0.612 | 0.613 | 0.613 | 0.623 | 0.628 |
| **MAEF-GO** | **0.635** | **0.636** | **0.639** | **0.648** | **0.652** |
| $F_{\max}$of CC ontology | | | | | |
| Model | <30% | <40% | <50% | <70% | <95% |
| DeepGO | 0.583 | 0.584 | 0.585 | 0.589 | 0.595 |
| DeepFRI | 0.604 | 0.606 | 0.607 | 0.606 | 0.613 |
| HEAL | 0.684 | 0.682 | 0.684 | 0.686 | 0.687 |
| TAWFN | 0.695 | 0.692 | 0.692 | 0.69 | 0.693 |
| **MAEF-GO** | **0.724** | **0.723** | **0.725** | **0.719** | **0.720** |

Table S5. Smin of MAEF-GO and other methods on the PDBset test set at five homology thresholds.

| $S_{\min}$of MF ontology | | | | | |
| --- | --- | --- | --- | --- | --- |
| Model | <30% | <40% | <50% | <70% | <95% |
| DeepGO | 0.542 | 0.532 | 0.514 | 0.485 | 0.475 |
| DeepFRI | 0.505 | 0.500 | 0.485 | 0.453 | 0.436 |
| HEAL | 0.391 | 0.388 | 0.372 | 0.351 | 0.341 |
| TAWFN | 0.373 | 0.365 | 0.354 | 0.336 | 0.326 |
| **MAEF-GO** | **0.348** | **0.340** | **0.329** | **0.308** | **0.298** |
| $S_{\min}$of BP ontology | | | | | |
| Model | <30% | <40% | <50% | <70% | <95% |
| DeepGO | 0.596 | 0.595 | 0.589 | 0.575 | 0.573 |
| DeepFRI | 0.570 | 0.564 | 0.561 | 0.545 | 0.541 |
| HEAL | 0.522 | 0.524 | 0.521 | 0.512 | 0.510 |
| TAWFN | 0.494 | 0.494 | 0.495 | 0.486 | 0.483 |
| **MAEF-GO** | **0.474** | **0.474** | **0.472** | **0.464** | **0.461** |
| $S_{\min}$of CC ontology | | | | | |
| Model | <30% | <40% | <50% | <70% | <95% |
| DeepGO | 0.554 | 0.556 | 0.551 | 0.546 | 0.540 |
| DeepFRI | 0.533 | 0.527 | 0.523 | 0.525 | 0.524 |
| HEAL | 0.461 | 0.461 | 0.458 | 0.461 | 0.458 |
| TAWFN | 0.451 | 0.456 | 0.454 | 0.456 | 0.454 |
| **MAEF-GO** | **0.423** | **0.422** | **0.419** | **0.426** | **0.426** |

Fig. S1. AUPR of different methods on PDBset test set over different IC (information content) range.

Table S6.Performance of MAEF-GO on AFset and ExtSet test sets under different training data settings.

| Score | MAEF-GO (AFset) | | |  | MAEF-GO-Ext (AFset) | | |  | MAEF-GO (ExtSet) | | |
| --- | --- | --- | --- | --- | --- | --- | --- | --- | --- | --- | --- |
|  | **MF** | **BP** | **CC** |  | **MF** | **BP** | **CC** |  | **MF** | **BP** | **CC** |
| $F_{\max}$ | 0.537 | 0.495 | 0.628 |  | 0.534 | 0.492 | 0.630 |  | 0.519 | 0.486 | 0.613 |
| AUPR | 0.543 | 0.245 | 0.323 |  | 0.559 | 0.246 | 0.322 |  | 0.546 | 0.235 | 0.319 |

MAEF-GO (AFset): MAEF-GO model evaluated on the AFset test set.

MAEF-GO-Ext (AFset): MAEF-GO model trained with an expanded training set including 10,269 low-homology proteins, evaluated on the AFset test set.

MAEF-GO (ExtSet): MAEF-GO model evaluated on the ExtSet test set, which consists of the 10,269 low-homology proteins.

**4. Interpretability of MAEF-GO**

*
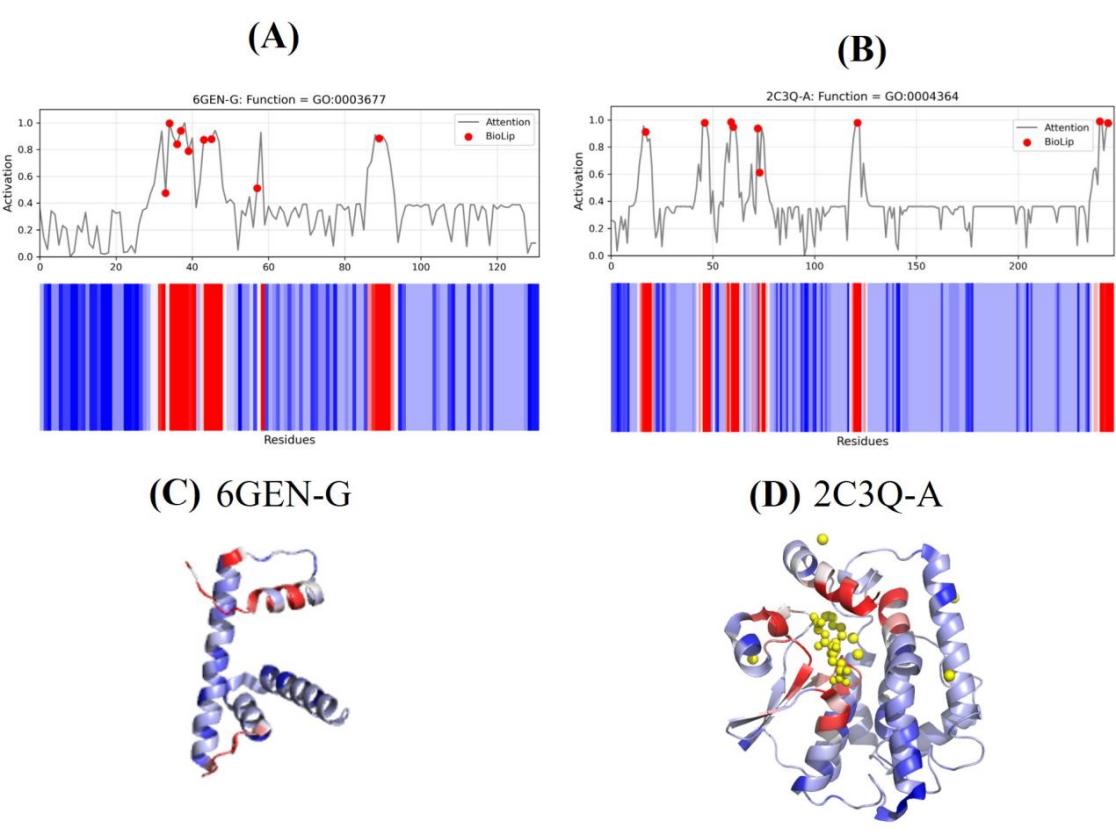
*

Fig. S2. (A) and (B): Visualization of residue attention weights; (C) and (D) Projection of heatmaps onto the 3D structure of the corresponding protein.
